# Cytogenetic aberrations in adult acute lymphoblastic leukemia—A population‐based study

**DOI:** 10.1002/jha2.300

**Published:** 2021-09-22

**Authors:** Emma Bergfelt Lennmyr, Marie Engvall, Gisela Barbany, Linda Fogelstrand, Hanna Rhodin, Helene Hallböök

**Affiliations:** ^1^ Department of Medical Sciences Uppsala University Uppsala Sweden; ^2^ Department of Immunology, Genetics and Pathology Uppsala University Uppsala Sweden; ^3^ Department of Molecular Medicine and Surgery Karolinska Institute Stockholm Sweden; ^4^ Department of Clinical Chemistry Sahlgrenska University Hospital Gothenburg Sweden; ^5^ Department of Laboratory Medicine, Institute of Biomedicine University of Gothenburg Gothenburg Sweden

**Keywords:** ALL, clinical cytogenetics, clinical hematology, cytogenetics of leukemia

## Abstract

Cytogenetic aberrations are recognized as important prognostic factors in adult acute lymphoblastic leukemia (ALL), but studies seldom include elderly patients. From the population‐based Swedish ALL Registry, we identified 728 patients aged 18–95 years, who were diagnosed with ALL 1997–2015 and had cytogenetic information. Registry data were complemented with original cytogenetic reports.

*BCR‐ABL1* was the most recurrent aberration, with a frequency of 26%, with additional cytogenetic alterations in 64%. *KTM2A* rearrangement was the second most frequent aberration found in 7%. Low hypodiploidy‐near triploidy and complex karyotype had negative impact, while t(1;19);*TCF3‐PBX1* showed positive impact on overall survival. However, after correction for age only complex karyotype remained significant.

## 1 INTRODUCTION

Information regarding cytogenetic aberrations has been recognized as important for prognosis in acute lymphoblastic leukemia (ALL) for decades [1, 2]. Thus, to genetically characterize ALL at diagnosis is mandatory and has implications for choice of treatment [3]. Still, genetic reference studies in adult ALL are infrequent and often derived from clinical trials with a corresponding selection bias excluding or not completely representing elderly ALL patients. In 2010, Moorman *et al*. [4] described the genetic findings in a cohort of 349 patients with adult ALL diagnosed in the northern part of England but apart from this, population‐based studies are scarce.

The aim of this study was to describe the panorama of genetic aberrations found in adult ALL, and their association with prognosis in a population‐based setting.

## 2 MATERIALS AND METHODS

The Swedish ALL Registry harbors a dual reporting system, where both pathologists and clinicians are required to report all adult patients who are diagnosed with ALL [5]. In 2019, we published results on outcome, including frequency of *BCR‐ABL1* and treatment, from the registry of 933 patients diagnosed from 1997 to 2015 [5]. In this paper, we have scrutinized the reported cytogenetic information in the registry for this cohort and supplemented the registered information with the original cytogenetic reports retrieved from the six cytogenetic facilities located at the University Hospitals in Sweden.

Definitions of genetic aberrations, methodology, and statistical analysis used are described in Supporting Information. For all comparisons, normal karyotype with ≥20 analyzed metaphases was used as comparator. The study was conducted in accordance with the Declaration of Helsinki and the regional ethical committee in Uppsala approved to the study (Dnr 2016/349). Vital status was followed until May 2018.

## 3 RESULTS

The median age of the 933 patients reported to the registry was 53 years (range 18–95). Details regarding immunophenotype and treatment are described in the previous registry publication [5]. Out of the 933 patients, 205 did not have any reported cytogenetic analysis from diagnosis. These patients had a median age of 66 years (range 18–95); 53% were male and median date of diagnosis was May 2003. Twelve had some genetic analysis performed but not at the time of diagnosis and 23 had an inconclusive or failed analysis. One patient had been diagnosed abroad and for the remaining 170 patients no cytogenetic analysis was done at all.

The 728 patients with any cytogenetic analysis had a median age of 50 years (range 18—90); 57% were male and the median date of ALL diagnosis was June 2008. The immunophenotypes were distributed in B‐ALL 72%, T‐ALL 15%, Burkitt leukemia 4%, and ALL NOS 9%. Sixty‐four patients with a sole negative test for *BCR‐ABL1* and/or *KTM2A‐*rearrangement (*KMT2A*‐r) were included only in the frequency analysis for these specific aberrations. For the remaining cohort of 664 patients, the main cytogenetic abnormalities are described in Table 1, divided by immunophenotype and age. According to the clinical registry report, remission inducing therapy was intended in 627 (94%) of the patients.

**TABLE 1:** Patient characteristics and cytogenetic findings in 664 patients

N (%)	All	B‐ALL	T‐ALL	Burkitt‐leukemia	NOS	18–30 years	31–50 years	51–70 years	≥71 yeras	Median age^e^ (range)	Males N^e^ (%)	Median WBC^f^ (range)
Cohort with cytogenetic analysis^a^	664	472	99	28	65	152	181	228	103	51 (18–90)	381 (57)	14 (0.5–904)
*BCR‐ABL1* ^b^	176 (26)	167(34)	1	–	8 (13)	17 (11)	65 (33)	72 (31)	22 (22)	52 (19–87)	92 (52)	**29 (0.7–477)** **
Normal karyotype^c^ ≥20 metaphases analyzed	83 (12)	52 (11)	22 (22)	3 (11)	6 (9)	20 (13)	18 (10)	34 (15)	11 (11)	53 (18–78)	52 (63)	10 (1–212)
No aberration, <20 metaphases analyzed	39 (6)	27 (6)	9 (9)	2 (7)	1	9 (6)	9 (5)	14 (6)	7 (7)	56 (18–90)	25 (64)	9 (1–26)
*KTM2A‐r* ^b^	46 (7)	36 (8)	2 (2)	–	8 (12)	7 (5)	15 (8)	16 (7)	8 (9)	54 (18–75)	**17** **(37)** **	**114 (5–904)** **
t(8;14)^d^	30 (4)	5 (1)	2 (2)	19 (68)	4 (6)	5 (3)	10 (6)	9 (4)	6 (6)	53 (19–81)	23 (77)	11 (5–120)
HeH	28 (4)	22 (5)	–	1	5 (8)	15 (10)	2 (1)	7 (3)	4 (4)	28 (18–76)	16 (57)	**4 (0.6–24)** **
Ho‐Tr	27 (4)	23 (5)	1	–	3 (5)	3 (2)	4 (2)	13 (6)	7 (7)	**64 (18–80)** *	17 (63)	6 (1–33)
Complex karyotype	22 (3)	13 (3)	2 (2)	2 (7)	5 (8)	2 (1)	4 (2)	10 (4)	6 (6)	**62 (22–80)** *	**8 (36)** *	4 (0.7–43)
Tetraploidy	8 (1)	2 (0,4)	3 (3)	–	3 (5)	2 (1)	4 (2)	2 (1)	–	37 (18–65)	3 (38)	4 (2–71)
t(1;19);*TCF3‐PBX1*	7 (1)	6 (1)	–	–	1	3 (2)	1	2 (1)	1	49 (22–76)	**1 (14)** *	20 (10–28)
Other	198 (30)	119 (25)	57 (58)	1	21 (32)	69 (45)	49 (27)	49 (22)	31 (30)	40 (18–87)	127 (64)	9 (0.5–395)

Abbreviations: HeH, high hyperdiploidy; Ho‐Tr, low hypodiploidy‐near triploidy; NOS, not otherwise specified; WBC, white blood cell count.

* *p *< 0.05

** *p *< 0.01.

^a^ Excluding patients with negative FISH or PCR only examining *BCR‐ABL1* or *KTM2A*‐r.

^b^ Frequency of adequately examined patients (as described in Supporting Information); *BCR‐ABL1, n* = 688 and *KTM2A‐r, n* = 647 patients.

^c^ Normal karyotype only including ≥20 metaphases.

^d^ The group t(8;14) also includes t(2;8) and t(8;22).

^e^ Comparison with normal karyotype as control.

^f^ WBC only available for patients with diagnose 2007 – 2015. Comparison with normal karyotype as control.

The translocation t(9;22)(q34;q11); *BCR‐ABL1* was the most frequent cytogenetic aberration, detected in 26% of investigated patients and predominantly associated with the B‐ALL phenotype (Table 1). In patients with confirmed *BCR‐ABL1* fusion (*n *= 124), 72% harbored the *BCR‐ABL1* minor transcript (p190) (median age 57 years [range 19–87]), and 27% the *BCR‐ABL1* major (p210) transcript (median age 45 years [range 21–76]). One patient had no specified transcript.

Additional cytogenetic aberrations (ACA) to *BCR‐ABL1* were frequent and found in 64% of the patients, 19% had no ACA, and 17% were not examined with anything but a targeted investigation or had a normal karyotype with ≥20 metaphases (*n *= 6). The most common ACA was an additional Philadelphia chromosome +der(22)(t(9;22) (*n *= 32), followed by –7 (*n *= 14) and +8 (*n *= 11). Of these, eight had a combination of +der(22)(t(9;22) and −7/+8. Having a p190 versus p210 transcript did not significantly impact overall survival (OS), nor did the presence of an ACA, or any of the three most frequent ACA versus no detected ACA (data not shown, analysis performed with correction for age).

*KTM2A*‐r was the second most frequent aberration, found in 46 out of 647 investigated patients (7%). Information about the fusion partner gene was available for 43 of 46 patients and the majority (*n *= 33, 77%) had a *KTM2A‐AFF1* fusion arising from the t(4;11)(q21;q23) translocation (median age 48, range 18–74 years). The translocation t(11;19)(q23;p13), where *KMT2A* can have several fusion partners, was found in six (14%) patients (median age 63, range 39–73 years). *CBL* and *ARHGEF12* as partner genes to *KTM2A* as well as t(1;11)(p32;q23) and t(4;11;15) were found in one case each. No difference was observed regarding OS between t(4;11) and t(11;19), analysis made with and without correction for age.

The 5‐year OS and hazard ratios (corrected for age) are shown in Table 2. Among the “other” cytogenetic aberrations, dic(7;9)(p11‐13;p11) was found in three B‐ALL cases aged 51–73 years. Interesting, all three were alive at follow‐up, 7–15 years after diagnosis.

**TABLE 2:** Survival for different cytogenetic groups

	5‐year OS (95%CI)	*p* (log‐rank)	HR (95% CI)^a^	*p*
*BCR‐ABL1*	41 (33–48)%	0.86	0.95 (0.68–1.31)	0.75
Normal karyotype, ≥20 metaphases analyzed	43 (32–54)%	Control	Control	
No aberration, <20 metaphases analyzed	37 (22–52)%	0.50	1.20 (0.76–1.92)	0.43
*KTM2A*‐r	36 (22–51)%	0.09	1.40 (0.90–2.17)	0.13
t(8;14)^b^	43 (26–61)%	0.74	1.13 (0.67–1.91)	0.65
HeH	49 (30–68)%	0.40	1.36 (0.75–2.46)	0.31
Ho‐Tr	18 (4–33)%	**<0.01**	1.55 (0.93–2.58)^c^	0.09
Complex karyotype	18 (2–34)%	**<0.01**	**2.05 (1.2–3.49)** ^d^	**<0.01**
Tetraploidy	38 (4–71)%	0.57	1.98 (0.78–5.07)	0.15
t(1;19);*TCF3‐PBX1*	86 (60–100)%	**0.03**	0.16 (0.02–1.14)	0.07
Other	45 (38–52)%	0.49	1.07 (0.77–1.48)	0.70

Abbreviations: CI, confidence interval; HeH, high hyperdiploidy; Ho‐Tr, low hypodiploidy‐near triploidy; HR, hazard ratio.

^a^ Cox regression adjusted for age.

^b^ The group t(8;14) also includes t(2;8) and t(8;22).

^c^ Ho‐Tr HR (95% CI) 1.98 (1.20–3.26) *p *< 0.01 uncorrected for age.

^d^ Complex karyotype HR (95% CI) 2.19 (1.29–3.70) *p *< 0.01 uncorrected for age.

t(1;19);*TCF3‐PBX1* was confirmed in seven patients, with a female predominance. The OS was higher compared to normal karyotype but when corrected for age it lost significance. Six of the seven patients were alive at follow, 2–10 years after diagnosis.

Patients with high hyperdiploidy (HeH) ALL had lower WBC at diagnosis. When corrected for age, HeH did not appear as a favorable cytogenetic risk group, with a hazard ratio of 1.36 for OS (n.s.). Complex karyotype and low hypodiploidy‐near triploidy (Ho‐Tr) correlated with inferior OS as well as with a high median age. After correction for age, only complex karyotype remained as a negative factor for OS. In the group with complex karyotype, potential loss of the *TP53* gene was found in five of 22 cases.

## 4 DISCUSSION

Cytogenetic aberrations are important for risk classification in adult ALL, with *BCR‐ABL1* being the most frequent aberration. Cytogenetic alterations affect the choice of treatment, which in Sweden is stipulated by national guidelines. In our study, the majority of *BCR‐ABL1* cases harbored ACA, with an additional derivative chromosome 22, −7, and +8 being most common, as previously reported [6]. The impact of ACA reported in some studies [7, 8, 9, 10] was not confirmed here, or in the study by Moorman et al. [6].

*KTM2A*‐r is commonly recognized as a negative prognostic factor [11], but the negative impact on prognosis is not always retained in multiple variable analysis correcting for other factors [6, 12]. We did not find a difference in outcome for patients with KTM2A‐r compared to those with normal karyotype, nor was there a difference in outcome for patients with different KTM2A‐r subtypes. In Sweden, the chemotherapy regimen has included high‐dose cytarabine, which is suggested beneficial for *KTM2A*‐r in children [13]. In addition, allogeneic hematopoietic stem cell transplantation has been recommended for fit patients for the main part of the period, which might have influenced outcome. Yet, the relatively low number of patients could also have hampered the analyses.

HeH is well known as a favorable prognostic factor for children with ALL, but few data are available for adults. In our study, there was a trend toward younger age and lower WBC, but when correcting for age, HeH was not a favorable factor in adult ALL, confirming previous reports [6, 11, 14]. Atypical chromosome gains compared to classical pediatric HeH [11] and the presence of two or more additional structural abnormalities [14] has previously been correlated to adverse survival in adult cohorts. This could indicate that pediatric and adult HeH are separate entities.

Ho‐Tr and t(1;19);*TCF3‐PBX1* impacted OS in the univariable analysis but lost significance when corrected for age. The groups included very few patients, precluding any firm conclusions.

The only negative cytogenetic risk factor that remained after correction for age was complex karyotype. This has been open to debate since the study by Moorman et al. [6] showed inferior survival, later confirmed by some [12] but not all study groups [11, 15]. One possible explanation could be specific alterations such as loss of p53; however, the frequency was too low to solely explain the impact of complex karyotype on prognosis. Complex karyotype has not been considered a high risk factor in Sweden, and thereby allografting has not been recommended upfront in cases with good initial response. In this material, it was done as upfront treatment for only eight of the 22 patients harboring a complex karyotype.

The strength of our study is that we report the frequency of genetic alterations found in adult ALL in a population‐based manner with no upper age limit. The weakness is associated with the limited details concerning clinical data in the registry. Also, diagnostic procedures have rapidly evolved and cytogenetic diagnostics have reached standards that were not available for patients in the early part of the study period.

We conclude that complex karyotype is a previously debated negative prognostic factor in adult ALL. With treatment according to national guidelines, no other aberration including *BCR‐ABL1*, *KTM2A*‐r, or HeH impacted survival. Incorporating complex karyotype in future risk stratification should therefore be considered.

## AUTHOR CONTRIBUTIONS

Emma Bergfelt Lennmyr and Helene Hallböök designed the study, analyzed statistics, and wrote the paper. Marie Engvall, Gisela Barbany, and Linda Fogelstrand evaluated and classified cytogenetic data and contributed to the writing of the paper. Hanna Rhodin collected and classified data. All authors critically reviewed and approved the final draft.

## CONFLICT OF INTEREST

The authors declare no conflict of interest.

## Supporting information

Supporting Information
