# Supplementary material for: Cytogenetic aberrations in adult acute lymphoblastic leukemia—A population‐based study
Source: EJHaem. 2021 Sep 22;2(4):813–7. doi: 10.1002/jha2.300 (PMC9175914; doi:10.1002/jha2.300)
Supplement: Supplementary file 1 — Supporting Information [file JHA2-2-813-s001.docx]

Supplemental I

During the study period (1997 and 2015), the methodology to genetically characterize leukemia samples gradually shifted from mainly using conventional chromosome banding analysis to include multiple methods such as FISH, microarray and other molecular techniques. Combined data from different modalities were accepted when performed and interpreted at the responsible diagnostic facility and later centrally reviewed by a cytogeneticist.

Patients who were only investigated with a single technique e.g. FISH, PCR or Southern blot analysis for detection of particular aberrations such as *BCR*-*ABL1* and/or *KTM2A*-rearrangements were accounted for when reported positive in either analysis. However, they were regarded as insufficient data in the overall evaluation, if negative. For calculation of the frequency of *BCR*-*ABL1* and/or *KTM2A*-rearrangements, normal karyotype was considered as insufficient data if <20 metaphases were analyzed, as were cases with karyotypes without clonal abnormality (as high hyperdiploidy and low hyperdiploidy-near triploidy) if no targeted analysis was performed. Loss of chromosome Y was not considered as abnormal.

High hyperdiploidy was defined as 51-65 chromosomes and low hyperdiploidy-near triploidy as 30-39 or 60-78 chromosomes. Complex karyotype was defined as five or more chromosomal abnormalities in the absence of an established cytogenetic subgroup. The group named t(8;14) included the aberrations t(8;14)(q24;q32), t(2;8)(p12;q24) and t(8;22)(q24;q11). Cases with confirmed *IGH*-rearrangements other than 8q24 were included in the group named “other” in absence of another established subgroup.

Statistical analyses were performed using the IBM SPSS software, version 27. Mann-Whitney U test was applied to compare continuous variables and Fisher’s exact test to compare categorical variables. Overall survival was estimated using the Kaplan-Meier method with log-rank test to compare groups. Uni- and bivariable Cox Regression analyses were used to determine hazard ratios for overall survival.
